# Tumor-associated macrophage-related strategies for glioma immunotherapy

**DOI:** 10.1038/s41698-023-00431-7

**Published:** 2023-08-19

**Authors:** Fansong Tang, Yuelong Wang, Yunhui Zeng, Anqi Xiao, Aiping Tong, Jianguo Xu

**Affiliations:** 1 Department of Neurosurgery, West China Hospital, Sichuan University, Chengdu, Sichuan Province China; 2 State Key Laboratory of Biotherapy and Cancer Center, West China Hospital, and Collaborative Innovation Center of Biotherapy, Sichuan University, Chengdu, Sichuan China

**Keywords:** Cancer immunotherapy, CNS cancer

## Abstract

High-grade glioma is one of the deadliest primary tumors of the central nervous system. Despite the many novel immunotherapies currently in development, it has been difficult to achieve breakthrough results in clinical studies. The reason may be due to the suppressive tumor microenvironment of gliomas that limits the function of specific immune cells (e.g., T cells) which are currently the primary targets of immunotherapy. However, tumor-associated macrophage, which are enriched in tumors, plays an important role in the development of GBM and is becoming a research hotspot for immunotherapy. This review focuses on current research advances in the use of macrophages as therapeutic targets or therapeutic tools for gliomas, and provides some potential research directions.

## Introduction

Glioma is the most common CNS malignancy in adults with a global annual incidence of 5–6 per 100,000 people and a highly heterogeneous and aggressive nature^1^. And glioblastoma, the most lethal glioma, accounts for 70–75% of all diffuse glioma diagnoses. Despite the availability of conventional treatments including surgery, radiotherapy, and chemotherapy, the median survival of patients is only 14–17 months^2^. As a result, the development of new therapies is very crucial, for example, targeted therapy, immunotherapy and electric field therapy.

Studies over the past two decades have revealed that tumor microenvironment (TME) is a pivotal determinant of tumor behavior, and is responsible for tumor progression and metastasis^3^. In addition, the discovery of intracranial lymphatic vessels has led to an increased recognition of the importance of immune cells in brain tumors, challenging previous assumptions about brain tolerance and immune privilege^4^. Glioma is characterized by a highly suppressive and unique “cold” immune microenvironment, which includes tumor cell-derived immunosuppressive factors, exhausted cytotoxic T lymphocytes (CTLs), Treg cells, and downregulated MHC and self-presentation^5^. Although T-cell associated therapies, such as chimeric antigen receptor T (CAR-T) cell therapy, or immune checkpoint inhibitors (ICI) targeting programmed death-ligand 1 (PD-L1) and cytotoxic T-lymphocyte-associated protein-4 (CTLA-4), have shown promise in treating extracerebral tumors^6^, the weak lymphocyte responsiveness of GBM’s TME limits their efficacy in GBM immunotherapy^7–9^.

**Fig. 1:**
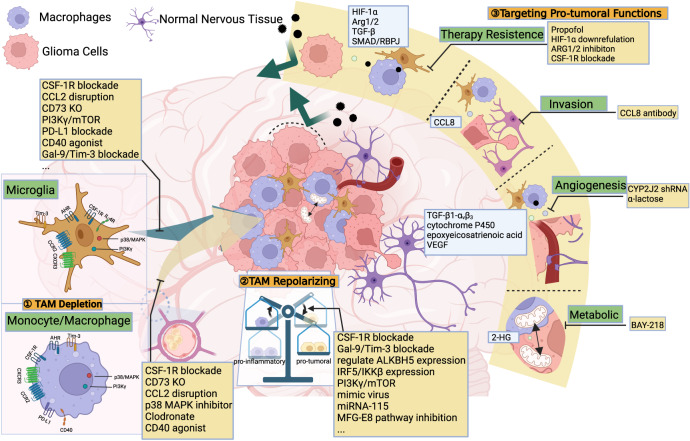
Three major classes of TAM-targeted glioma therapies. TAM mainly originates from microglia in the brain and peripheral-derived monocytes, and its activation types contribute to a suppressive immune microenvironment and thus results in various tumor biological behavior. As a result, TAM-targeted therapy mainly targets its recruitment, polarization, and its multiple function in tumor behavior. TAM tumor-associated macrophage, CCL chemokine ligand, CYP cytochrome P450, CSF colony-stimulating factor, VEGF vascular endothelial growth factor, HIF-1 hypoxia-inducible factors-1 (Created with BioRender.com).

**Fig. 2:**
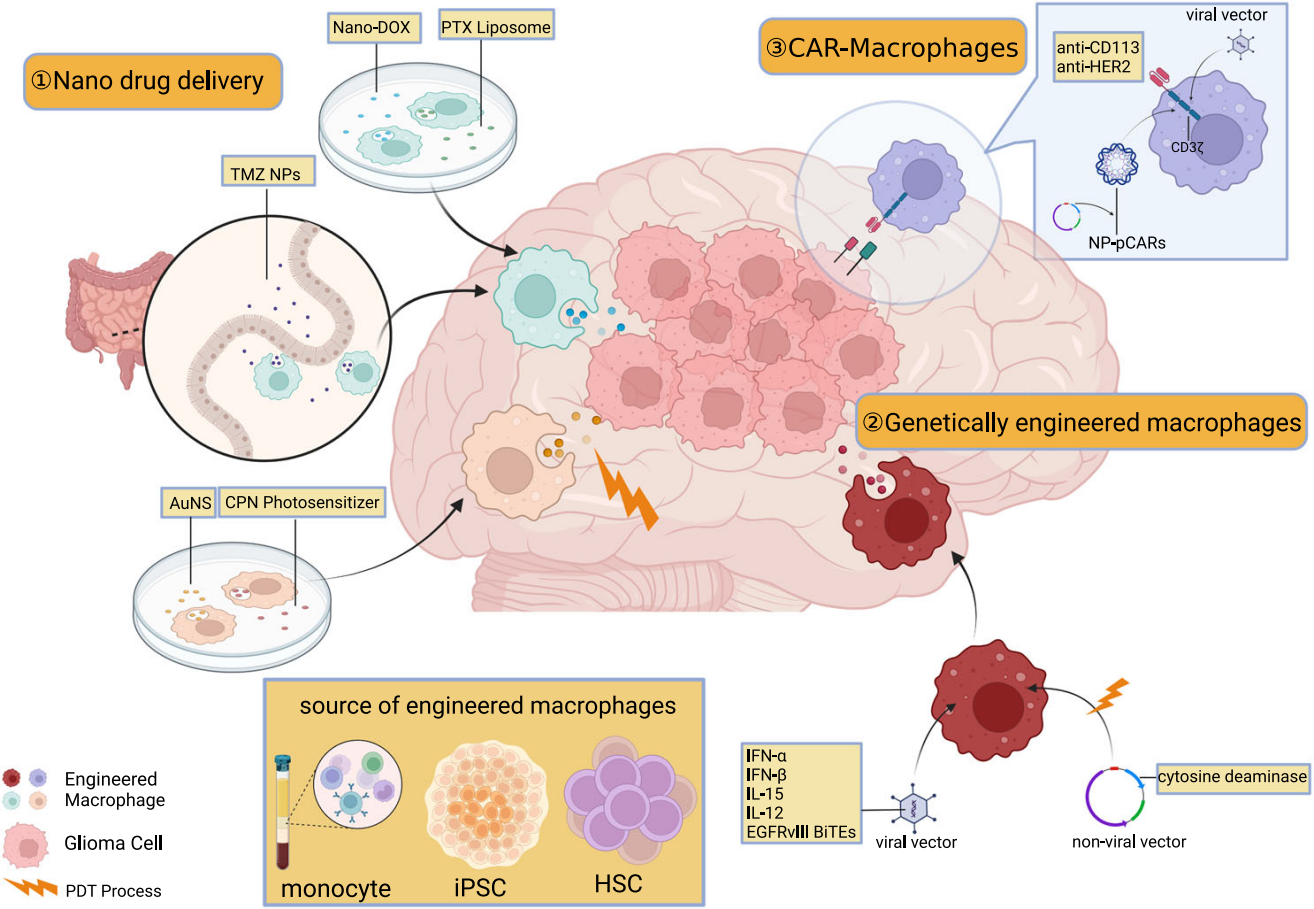
Exploiting engineered macrophages for glioma therapy. Because of its tumor tropism, macrophages are ideal vehicle to carry therapeutic matters to tumor site. General payloads include conventional chemotherapy drugs, medium of the physical treatments, and genes of immunotherapeutic biomolecules. Latest technology also enables loading of CARs on macrophages, potentially breaking through the limitations of CAR therapy in solid tumors. pCAR chimeric antigen receptor plasmid, PDT photodynamic therapy, CPN conjugated polymer nanoparticles, TMZ NP temozolomide nanoparticle, DOX doxorubicin, PTX paclitaxel, CD cytosine deaminase, IFN interferon, NS nanoshells, EGFRv epidermal growth factor variant, BiTE bispecific T-cell engagers (Created with BioRender.com).

Given the limited success of single lymphocyte-related therapies, investigating other immune cells within the GBM microenvironment may hold the key to effective immunotherapy^5^. Tumor-associated macrophages (TAMs) comprise a significant portion of the tumor mass, accounting for 30–50% of the cells^10^, with ~15% derived from intrinsic microglia and 85% recruited from peripheral-derived monocytes by tumor-derived chemokines^11^. TAMs can be classified into several groups based on their ontogeny, surface protein markers, and transcriptomic data^12^. Some TAMs promote tumor angiogenesis, immune evasion, and tumor proliferation^13–15^, leading to higher tumor grade, poorer prognosis, and increased treatment resistance, while others display antitumor activity^16^. In light of these findings, therapeutic strategies targeting pro-tumoral TAMs can be designed, such as TAM depletion, re-educating and suppression of their pro-tumor functions (Fig. 1). In addition, recruited monocyte/macrophages can be manipulated as tools for biological therapies by exploiting their ability to transfer drugs or therapeutic genes (Fig. 2).

## Macrophages as targets in glioma therapy

Therapies targeting TAMs have been performed intending to inhibit their pro-tumor function. Since GBM-derived chemokines such as GDNF (glial cell-derived neurotrophic factor), GM-CSF (granulocyte-macrophage colony-stimulating factor), and CCL2 (chemokine ligand), have been proven to be involved in TAM recruitment and polarization^17^, classical methods are to deplete TAMs and reshape TAM population by targeting these molecules^18^ (Table 1). With a deeper understanding of heterogeny and various roles of TAMs in tumor development, therapies targeting specific tumor-promoting mechanisms of TAM are also being developed.

**Table 1:** Information about Targeted therapy for tumor-associated macrophages.

Strategy	Method	Mechanism	Medication route	Effect on macrophages	Effect on tumor	Reference
TAM depletion	PLX3397+cediranib	CSF-1R + VEGFR2 inhibition	Oral	Reshape TAMs and reduce angiogenesis function	Decrease vessel density and cell proliferation	^24^
	BLZ945 + OSI906	CSF-1R + IGF-1R inhibition	Oral	Downregulate M2-like gene and IGF in BMDMs	Overcome tumor resistance to CSF-1R blockade therapy	^22^
	NE-siRNA-CD73	CD73 inhibition	Nasal	Reduce Tregs, microglia, and macrophages	Induce tumor cell apoptosis	^25^
	CCX872/Anti-PD-1	CCL2/CCR2 axis disruption	Oral	Reduce tumor-associated MDSCs and activate T cells	Enhance Anti-PD-1 therapy and prolong survival	^29^
	Celecoxib	CCL2 and CXCL10 inhibition	Oral	Reduce microglia and macrophages	Induce apoptosis, inhibits GSC viability	^28^
	CCR2 knockout	CCL2/CCR2 axis disruption	–	Decrease TAM infiltration	Enhance tumor proliferation and vascular integrity	^30^
	TG100–115	PI3Kγ inhibition	Intraperitoneal	Suppresses microglia/TAM accumulation and secretion of IL-11	Suppress tumor genesis and TMZ resistance	^35^
	lncRNA-miRNA- ALKBH5	Regulate ALKBH5 expression	Subcutaneous	Reduce M2-like cell infiltration	Suppress tumor proliferation	^33^
	CDX-LIPO	Targeting PI3K/mTOR	Intravenous	promote M1-like polarization	Induce autophagy and immunogenic cell death	^36^
	LY2228820	p38 MAPK inhibitor	Intraperitoneal	Inhibit macrophage aggregation	Suppress tumor proliferation	^37^
TAM re-education	Anti-PD-L1+radiation	Inhibit PD-L1 pathway+abscopal response	–	Activate macrophages, enhance phagocytosis	Prolong survival	^46^
	PD-L1 knockout	Inhibit PD-L1 pathway	–	Upregulate M1-like and downregulate M2-like populations	Inhibits proliferation and invasion	^44^
	Nivolumab/Bevacizumab	Inhibit PD-L1 pathway	Oral	Regulate TAM polarization	Prolong survival	^43^
	α-lactose	Gal-9/ Tim-3 blockade	Intraperitoneal	Inhibit M2-like polarization	Inhibit angiogenesis and tumor proliferation	^50^
	Anti–Tim-3 antibody	Gal-9/ Tim-3 blockade	Intravenous	Inhibit M2-like polarization	Inhibit angiogenesis and tumor proliferation	^50^
	*LGALS9*- targeted shRNAs	Gal-9/ Tim-3 blockade	In vitro	Inhibit M2-like polarization	Inhibit angiogenesis and tumor proliferation	^50^
	mRNA-NPs	IRF5/IKKβ expression	Intravenous	increase M1-like cell and decrease M2-like cells	Suppressed tumor progression and prolong survival	^52^
	CpG-Au-NPs	Enhance radio, re-polarize M2	Intratumoral	Increase M1-like cell and decrease M2-like cells	Enhance tumor killing of ICB and radiotherapy	^53^
	CD40 agonist+IL-6 neutralize +ICB	Macrophage activating	Orthotopic injection	Reverse immunosuppression, activate T cell	Sensitize tumor to ICB and prolong survival	^51^
	miRNA-155 Nanogel	Mimic virus	Intravenous	Increase M1-like cell and decrease M2-like cells	Inhibits tumor proliferation and prolong survival	^56^
	ITGB3 siRNA/anti‐MFG‐E8 antibody	MFG-E8 pathway inhibition	Transfect	Reduce M2-like and increase M1-like microglia	Suppress tumor growth	^57^
Targeting pro-tumoral function of TAMs	Propofol + TMZ	Downregulate HIF-1α	Intraperitoneal	Enhances macrophage infiltration and inflammation	Reduce drug resistance, promote apoptosis	^3^
	OAT-1746	Inhibiting ARG1/2	Oral	Transform TAM gene expression signature to a pro-inflammatory phenotype	Improves the efficacy of the PD-1 checkpoint inhibition	^63^
	PLX5622	CSF-1/CSF-1R inhibition	Oral	Prevents hippocampal-dependent memory deficits	–	^64^
	BLZ-945	CSF-1/CSF-1R inhibition	Oral	Reduce microglia and MDM populations, reduce peritumoral macrophages	Enhances initial response to radiotherapy, inhibits tumor recurrence	^66^
	CCL8 neutralized antibody	Inhibit ERK1/2	Subcutaneous	Decrease macrophage-derived CCL8	Decreases TAM-induced glioma invasion	^67^
	Integrin and TGFβ-R1 blockade	Inhibit Src-PI3K-YAP pathway	In vitro	Inhibit endothelial cells-macrophage interaction	Inhibit tumor angiogenesis	^68^
	CYP2J2 shRNA	Restrained the release of 11,12-EET	In vitro	Inhibit EET expression on M2-like microglia	Inhibit tumor angiogenesis	^69^
	α-lactose	Gal-9/ Tim-3 blockade	Intraperitoneal	Inhibit secretion of VEGF	Inhibit angiogenesis and tumor proliferation	^50^
	BAY-218	Inhibit AhR	Intravenous	Reverse immunosuppression enhance the function of macrophages	Decrease tumor proliferation and prolong survival	^70^

## TAM depletion and reduction

CSF-1/CSF-1R plays a central role in microglial and macrophage’s development and maintenance^19,20^. Unfortunately, depleting TAM by targeting CSF-1/CSF-1R has not been as effective in clinical trials as in preclinical trials^21^. Quail et al. found that resistance to CSF-1R inhibitors is linked to IGF-1 (insulin-like growth factor) and wound-associated signature from TAMs driven by IL-4/NFAT and Stat6 signaling. IGF-1 causes upregulation of the PI3K (phosphoinositide 3-kinase) pathway in tumor cells by binding to IGF-1R. Co-inhibition of IGF-1/PI3K and CSF-1R using OSI906/Linsitinib +BLZ945 was found to overcome tumor resistance to BLZ945 therapy, and extend median survival of mice from 13 days to 63 days^22^. In addition, GBM response to CSF-1R inhibition therapy may be related to the dictation of the TME by different tumor subtypes. Wang et al. conducted transcriptomic analyses to investigate the microenvironment of proneural, mesenchymal, and classical-type tumors and observed that mesenchymal GBM with NF1 mutation/loss displayed increased expression of macrophage-associated markers (Iba1, CD11b) and macrophage infiltration. Furthermore, they found that recurrent GBM had a higher tendency to transform into the mesenchymal subtype, which was associated with increased macrophage recruitment^23^. Rao et al. further investigated the differential response of proneural-like tumor and mesenchymal-like tumor to PLX3397. Single-Cell RNA-Seq reveals that the TAMs of PDGFB-driven proneural tumors is dominated by microglia and PLX3397 treatment can remodels the TAMs in this type of tumor by downregulating pro-tumor gene expression, leading to a favorable response to PLX3397 therapy. In contrast, TAMs from RAS-driven mesenchymal-like tumor exhibit pro-inflammatory and pro-angiogenic signaling, resulting in resistance to PLX3397 monotherapy or combination therapy with anti-PIK3 pathway^22^. Co-targeting of TAMs and angiogenesis using PLX3397+cediranib (a VEGFR2 inhibitor) could reduce tumor proliferation in mesenchymal-like tumors, but has a negative effect in proneural tumors^24^. These suggest that treatment targeting TAM needs to be more tailored to specific tumor subtypes.

CD73 is a promising target for GBM treatment, as this enzyme favors cancer progression and immune evasion by converting extracellular immuno-activating ATP into adenosine. To suppress CD73 expression, researchers have developed siRNA CD73-loaded cationic-Nano emulsion (NE-siRNA CD73R), which effectively reduced the population of Tregs (regulatory T cells), microglia, and macrophages in TME, and suppressed tumor growth^25^. This nanotechnology holds great potential for the targeted delivery of siRNA and improved GBM treatment. However, further investigations are needed to determine the optimal dosing and maintenance of siRNA in tumor tissues for gene therapy.

Targeting the CCR2/CCR2 axis can be an effective strategy for inhibiting the recruitment of TAMs. The chemokine CCL2, which is derived from the TME, is known to plays a crucial role in the migration and recruitment of blood-derived monocytes that contribute to the immunosuppressive TME and tumor progression^26,27^. Genetically interrupting CCL2 could prolonged the survival of mice bearing glioblastoma^11^. In addition, celecoxib has been found to reduce microglia and macrophages by inhibiting the expression of CCL2 and CXCL10 and^28^, in addition to its direct antitumor effects through the promotion of tumor cell apoptosis and regulation of cell cycle. CCR2 antagonists, such as CCX872, have also been shown to decrease the intratumoral macrophages population. When used in combination with PD-1 antagonists, CCX872 has been found to reduces myeloid-derived suppressive cells (MDSC) in tumor, enhance the activation of IFN axis and T cells, reduce exhaustion of T cells and improve tumor-killing ability, thereby benefiting the survival of mice bearing Kr158^29^. However, conflicting results have been reported in another research, where GL261 inoculation on *Ccr2*-deficient strain led to a 30% reduction of TAM, but also augmented tumor volumes^30^. Moreover, the clinical outcomes of this type of treatment are also not as good as in preclinical experiments suggest^31^. Such discrepancies may be attributed to the activation of other recruitment signaling pathways when CCR2 is deficient as the monocyte chemoattractant protein family forms a complex regulatory network. In addition, CCR2 expression in different tumor tissues may result in varying responses to CCR2 axis-targeted therapy^30^. Therefore, more preclinical studies are needed to characterize the chemotaxis of monocytes and verify the potential of this therapeutic target.

Some other therapeutic targets that inhibit TAM recruitment have been initially investigated. M^6^A demethylase ALKBH5 (AlkB homolog 5) highly expresses in GBM stem-like cells^32^. ALKBH5 expression promotes tumor proliferation, affects lymphocyte activation and infiltration, and is associated with M2-like macrophage infiltration^33^.

Kynurenine present in tumor-conditioned media activate AHR (aryl hydrocarbon receptor) in macrophages, resulting in increased expression of CCR2 as well as recruitment of TAM. AHR also drives CD39 expression, which can impair T-cell immune response. This suggests that they are both potential therapeutic targets for the TME^34^.

PI3Kγ plays a crucial role in promoting microglia chemotaxis and IL-11 secretion, which can in turn lead to tumorigenesis and resistance to TMZ by activating the STAT3-MYC axis in tumor cells. Targeting microglia by PI3Kγ inhibitor TG100–115 can suppress tumor genesis and therapy resistance, leading to a 6-day increase in the survival of mice bearing GL261^35^. Zheng et al. constructed honokiol (HNK), which can inhibit PIK3/mTOR (mammalian target of rapamycin), and the antitumor agent disulfiram/copper (DSF/Cu) into exosomes (CDX-LIPO) and targeted tumor capillary-specific Nicotinic acetylcholine receptors via ^D^CDX. This approach induces autophagy in tumor cells while promoting pro-inflammatory polarization of macrophages, activating multiple immune cells within the TME, regulating cellular metabolism and inhibiting the production of immunosuppressive lactate^36^. These findings suggest that PI3Kγ may be a promising therapeutic target for treating gliomas.

p38/MAPK pathway is associated with the recruitment of macrophage/microglia as well as higher PD-L1 expression in both tumor cells and TAMs. Inhibition of this pathway in combination with anti-PD-L1 antibody treatment has been shown to reduce infiltration of blood-derived CD45^high^/CD11b^+^ macrophages and decrease PD-L1 protein expression in microglia, leading to improved survival in GBM-bearing mice resistant to TMZ^37^. These findings suggest that targeting the p38/MAPK pathway in conjunction with PD-L1 blockade may be a promising therapeutic strategy for GBM.

Clodronate can be uptake by macrophages and induce TAM apoptosis in solid tissues in vivo and reduce macrophage-derived VEGF, thereby inhibiting tumor angiogenesis and tumor proliferation. Combining it with exosomes can increase tissue infiltration and reduce systemic toxicity, making it a potential immunotherapeutic tool^38,39^.

## TAM re-educating and repolarizing

Macrophages are highly plastic cells which can be activated and polarized by various factors including glioma cell-derived soluble molecules, non-coding RNAs, and factors induced by radiotherapy and chemotherapy^40^. Re-educating TAM to increase the proportion of pro-inflammatory subtype helps to remodel the immunosuppressive TME of gliomas, thereby potentially improving the efficacy of glioma treatment.

PD-L1 blockades has been widely used as a therapeutic strategy for many types of cancer, including GBM^41,42^. However, the exact mechanisms underlying therapeutic effects of PD-L1 blockade are not fully elucidated. Sydney R. Gordon has shed some light on the role of PD-1 in the recruitment and polarization of M2-like monocyte/macrophages showing that PD-1^+^ TAMs exhibit inferior phagocytic function, which can be reverted by PD-L1 blockade^43^. PD-L1 knockdown has also been found to upregulate M1-like and downregulate M2-like populations, which can prevent tumor cell invasion and migration^44,45^. Furthermore, combination therapy with anti-PD-L1 and radiation has been shown to increase the recruitment of immunosuppressive PD-L1^+^ monocytes in both radiated and non-radiated brain regions^46^. Additionally, anti-PD-1 antibodies can directly activate macrophages, resulting in an upregulation of cell cycle genes and cytokine production, a pro-inflammatory phenotype, and enhanced phagocytosis of tumor cells^46^.

In addition to its application in TAM depletion, CSF-1/CSF-1R also serves as a therapeutic target for TAM remodeling^47,48^. The aforementioned treatments targeting CSF-1R can modulate the polarization of TAM to some extent^22,24^. Another potential target for TAM modulation has been identified by Liu et al., who found that DExH-box helicase 9 enhances the expression of CSF-1 in tumor tissues by binding to transcription factor 12(TCF12), promoting the recruitment of TAMs and the expression of M2-related markers. Knockdown of this gene can inhibit macrophage recruitment and polarization and suppress tumor growth^49^. Thus, in CSF-1/CSF-1R inhibition therapy, TAM re-education and reduction act collectively to remodel the immunosuppressive TME of GBM^19,20,24^.

In *PTEN-*null GBM, the deficiency of *PTEN* gene leads to an increased secretion of Gal-9 (galectin-9) via AKT-GSK3β-IRF1 pathway. Gal-9 activate Tim-3 on macrophages and induce M2-like polarization and enhanced secretion of vascular endothelial growth factor A, resulting in tumor angiogenesis and tumor growth. Inhibition of different parts of this axis by α-lactose or Tim-3 antibody or LGALS9-targeted shRNAs can regulate TAM polarization and inhibit angiogenesis and tumor proliferation^50^.

CD40 costimulatory receptors on macrophages promote the effect of IL-12 and IFN and activate T-cell immunity. While IL-6 promotes the transition of macrophages to a pro-tumor phenotype and promotes the secretion of inflammatory suppressors such as IL-10 and TGF-β. However, Yang et al. found that IL-6 induces CD40 expression through Stat3 and HIF-1α. The combination of IL-6 neutralizing antibody and CD40 agonist enhances T-cell activation and ICB (immune checkpoint blockade) efficacy and extends survival from 21 to 37 days in GL261-bearing mice^51^.

Zhang et al. developed a mRNA based nanocarrier that stably express IRF5 (interferon regulatory factor) and its kinase IKKβ in TAMs by using a poly (β-amino ester) backbone linked to an anionic mRNA and decorated with Di-mannose moieties and polyglutamic acid on the surface. The approach leads to a shift towards M1-like phenotype, resulting in enhanced T-cell activation and recruitment^52^. Combination therapy with radiation resulted in a significant survival benefit in a preclinical model of glioma (25–52 days). CpG-decorated gold (Au) nanoparticles were also shown to re-educate TME. CpG is a toll-like receptor 9 agonist that can activate macrophages, promote M1-like polarization, and enhance phagocytosis and antigen presenting^53^. In addition, gold NPs serve as radio enhancers, making them ideal candidates for combination therapy with radiation^54^. These immunotherapies can promote abscopal response after radiotherapy and remodel the suppressive TAM recruited during radiotherapy^55^.

MiRNA-155 has been shown to downregulate the expression of anti-inflammatory proteins in microglia and macrophages, resulting in a pro-inflammatory phenotype. In addition, macrophage phagocytosis of viral particles can also promote the conversion to a pro-inflammatory phenotype. Gao et al. developed a viral gel by combining MiR-155 with nanohydrogels that are encapsulated with erythrocyte membranes and M2pep and HA2 peptides, which target tumor-promoting macrophages. This viral gel is stable in vivo and can be efficiently phagocytosed by macrophages, inducing differentiation to a pro-inflammatory phenotype and produce antitumor effects^56^.

In glioma, the presence of MFG-E8 (Milk fat globule EGF factor) derived from tumor cells promotes the ITGB3/STAT3 pathway, which leads to increased secretion of IL-4, ultimately resulting in the differentiation of microglia into a pro-tumor phenotype and upregulation of molecules such as TGF, IL-10, and CD206. Targeting MFG-E8 and its integrin β3 receptor inhibits TAM infiltration and reduce the expression of anti-inflammatory genes, reduces tumor size and improves tumor sensitivity to drugs^57^.

## Targeting the pro-tumor functionality of TAMs

TAMs perform a multitude of pro-tumor functions in the TME, including promoting therapy resistance, pro-tumor signaling, pro-angiogenesis and regulating energy metabolism^16^. Due to the diverse functionalities of TAMs, various mechanisms need to be investigated, and different therapies targeting have the potential to be developed^58^ (Fig. 1).

In the context of drug resistance, TAMs have been shown to contribute to the resistance against temozolomide (TMZ) and PD-L1 checkpoint blockade therapy. This resistance is associated with a more suppressive immune microenvironment and macrophage education^35,37,59^. Interestingly, propofol, a conventional anesthetic, has been found to downregulate HIF-1α expression, promote a pro-inflammatory phenotype and reduce drug resistance to TMZ^3^. As for PD-L1 checkpoint blockade, the pro-tumoral phenotype of TAMs is associated with ARG1 type of arginine metabolism^60,61^. MDSCs expressing ARG1 can alter T-cell activation and enhance tumor invasion^62^. However, inhibiting ARG1/2 by OAT-1746 unlocks antitumor response in myeloid cells, T cells and NK cells, reduce the expression of tumor-supportive gene in TAMs, and improves the efficacy of the PD-1 checkpoint inhibition^63^.

Tumor-associated macrophages (TAM) have been shown to play a role in various reactions following radiation therapy. For instance, memory deficits after whole-brain radiotherapy have been linked to post-radiotherapy monocyte recruitment, which can be mitigated by treatment with PLX5622 for 21 days. However, the exact role of macrophages in causing memory loss remains unclear^64^. In addition, Akkari et al. observed increased recruitment of TAMs as well as an increased proportion of mononuclear-derived macrophages in recurrent tumors after radiotherapy. Transcriptomic data from their study suggest that TAMs develop distinct genetic signatures after radiotherapy compared to untreated tumors. Specifically, both SMAD and RBPJ pathways were upregulated, while signatures associated with both types of TAM ontogeny remained relatively stable^65^. Targeting microglia and macrophages with BLZ945 not only enhances the efficacy of initial radiotherapy, but also inhibits tumor recurrence^66^.

As for enhancement of tumor aggression, CCL8 secreted by TAMs binds to CCR5 and CCR1 receptors, activating ERK1/2 phosphorylating signaling pathway and inducing pseudopodia formation of GBM cells. Blocking TAM-secreted CCL8 by neutralized antibody significantly decreases invasion of glioma cells^67^.

For tumor angiogenesis, M2-like cells have been demonstrated to contribute to glioma angiogenesis, which is prominently driven by the interactions between TGF-β1 and surface integrin (α_v_β_3_) interactions. Tuning cell-adhesion receptors using an integrin (α_v_β_3_)-specific collagen hydrogel can regulate inflammation-driven angiogenesis^68^. TAM cells also induce angiogenesis via CYP2J2 (cytochrome P450 2J2) and 11,12-EET (epoxyeicosatrienoic acid) expression. Thus, targeting CYP2J2 can reduce tumor angiogenesis and benefit glioma therapy^69^. Moreover, macrophage-derived VEGFA (vascular endothelial growth factor A) plays a crucial role in tumor angiogenesis in *PTEN-*null GBM, and α-lactose can attenuated tumor growth by inhibiting angiogenesis in this way^50^.

Regarding the altered tumor metabolism, tumor metabolites also educate macrophages in the tumor microenvironment to become a pro-tumor phenotype, and the products of TAM in turn lead to tumor growth^70^. One of the most well-studied metabolites is 2-HG, the product of mIDH (mutant isocitrate dehydrogenase). It causes downregulation of leukocyte chemotaxis, resulting in repression of the tumor-associated immune system^71^. In addition, IDH-dependent macrophage education, which is associated with decreased antigen presenting and CCL2 expressing, is related to a complex re-orchestration of tryptophan metabolism. Inhibition of AhR cellular chemoreceptor, a high regulated pathway receptor after TAM exposure to 2-HG, or tryptophan metabolism can reverse immunosuppression, enhance the immune function of macrophages, and prolong the survival of IDH1-mutant glioma-bearing mice in combination with PD-L1 blockade^70,72,73^.

Non-coding RNA and exosomes are crucial mediators of intercellular communication between tumor cells and TAMs^74–77^. They have been found to play important roles in shaping the tumor microenvironment. Moreover, they have been identified as promising targets for novel cancer therapies. For instance, non-coding RNAs can also be utilized for immunotherapy, such as controlling target gene expression and replication of oncolytic viruses^78^. Exosomes, on the other hand, can be used as vehicles for the delivery of therapeutic agents to target cells. Understanding the complex interplay between non-coding RNA, exosomes, and tumor-associated macrophages will provide valuable insights for the development of more effective cancer treatments.

With a deeper understanding of the role of TAM in tumor development, we can develop therapies that target more specified pro-tumor functions^22,79^. This will help to optimize treatment efficacy while minimizing potential side effects, ultimately improving patient outcomes.

## Macrophages as tools in glioma therapy

To achieve sufficient intratumoral accumulation, researchers exploit tumor-associated macrophages within the special tumor microenvironment to carry drugs or express genes^80,81^ (Fig. 2), for example, immune molecules and CARs (chimeric antigen receptor)^82^. Some details and features of these studies are presented as follows in Table 2.

**Table 2:** Information about engineered macrophages as glioma therapy tools.

Payload	Medium	Medicating route	Source of macrophages	Change of state of macrophage	Effects on tumor burden	Reference
Tie2-IFN-α	Lentivirus	Intravenous	Hematopoietic stem/progenitor cell	Activated and upregulated Iba1, Oas1a, TNF-α and IL1a/b	Inhibit angiogenesis, suppresses tumor growth and metastasis	^83^
Tie2-IFN-α	Lentivirus	Intravenous	Hematopoietic stem/progenitor cell	–	–	NCT03866109
IFN-β	Exo-AAV	Intravenous	Tumor stromal cell	Secrete IFN-β, and pro-inflammatory	10–20% tumor cell death	^84^
Cytosine Deaminase	Non-viral vector	In vitro	NR8383 cell line	Multi-drug resistance	Tumor cells decrease in hybrid cell monolayers	^93^
Cytosine Deaminase	–	Intratumoral	–	–	–	NCT04657315
EGFRvIII BiTEs	Lentivirus	Intratumoral	Monocyte-derived macrophages	Pro-inflammatory, also activate T cells	Prevented tumor growth for 36 days in the presence of T cells	^87^
EGFRvIII BiTEs	–	Intravenous	–	–	–	NCT04903795
IL-15	rAAV2	Intranasal	Microglia	Reduction of Arg1(+) pro-tumoral cells	Reduce tumor volume from 4.52 to 1.47 mm^3^	^59^
IL-12	Lentivirus	Intravenous	Monocyte-derived macrophages	Not changed after transduction, but turn pro-inflammatory in vivo	Slowed tumor growth and increased survival	^91^
DOX	Nanodiamonds	Intravenous	U937 cell line and peripheral monocyte	Increased expression of CD86(Pro-inflammatory)	Render damage-associated molecular patterns in tumor cells	^95^
TMZ	NPs	Oral	Intestinal macrophages	Become pro-tumoral by LPS and IFN stimulating	Reduce tumor volume and body weight loss	^99^
PTX	Liposomes	Intravenous	BV2 cell line	Pro-inflammatory	Lower doses and higher efficacy	^98^
Au	AuNS	Intracerebral	NR8383 cell line	Not mentioned	Reduce tumor volume	^101^
photosensitizer	CPNs	Intravenous	THP-1 and monocyte	Pro-inflammatory	No in vivo data	^102^
anti-HER2 CAR	Ad5f35	–	monocyte-derived macrophages	Pro-inflammatory	No data for GBM	^112^
anti-CD19 CAR	Lentivirus	–	Pluripotent stem cell	Pro-tumoral, but turn pro-inflammatory after exposure to tumor	No data for GBM	^129^
anti-CD133 CAR	pCARs(non-viral)	Intratumoral	Tumor stromal cell	Pro-inflammatory and gathered in tumor site	Tumor regression, inhibit tumor recurrence	^113^

## Engineered macrophages as therapeutic gene vector

Macrophages have been applied for the delivery and expression of the genes of biotherapeutic substances, of which one of the most classic is IFN (interferon). IFN has been used in tumor therapy since 1986 to modulate immunity and inhibit angiogenesis. Unfortunately, its clinical application is limited due to its short half-life and high toxicity. Thus, lentiviral vector transduced IFN-α monocytes, which selectively express IFN-α under the control of GBM-specific angiopoietin receptor *Tie2* promoter/enhancer elements and accumulate to tumor, were used as a vehicle for targeted delivery^83^. Because of the preferential activation of the Tie2 promoter in the TME, continuous, low-dose IFN-α would be released at the tumor site without inducing counterregulatory responses and systemic toxicity. IFN-α then stimulates and activates immune cells (e.g., macrophages, DCs, and T cells), inhibits angiogenesis, and suppresses tumor growth and metastasis. And a clinical study based on this technology is also underway (NCT03866109).

As for IFN-β, to exploit tumor stoma cells including TAMs in situ to secret antitumor agent IFN-β(interferon), AAV (adeno-associated virus) was injected intravenously with exosomes (exo-AAV) enhancing the ability to infect cells. The AAV encoding IFN-β was mediated by glioma stoma-specific promoter (GFAP for astrocyte and 5-NF for macrophages/microglia)^84^. Both types of cells can then secrete IFN-β, but the therapeutic effect of modified TAMs is weaker than that of modified astrocytes. The dilution of AAV vectors due to tumor growth may result in less effective gene expression in TAMs than in the more stable astrocytes, though AAV does effectively infect cells in CNS^85^. Besides, this study also mentioned the narrow therapeutic window of IFN-β. Therefore, the application of in situ genetic engineering requires the selection of a more persistent virus and a more refined TAM-specific promoter.

Macrophages have also been engineered to express BiTEs (bispecific T-cell engager) to facilitate the interactions of T cells and tumor cells via binding of a CD3ε and GBM-specific EGFRvIII (epidermal growth factor variant)^86^. Human monocyte-derived macrophages were transduced with lentivirus and secreted BiTEs in EGFRvIII expressing tumor site. The method resulted in the enduring expression of BiTEs, upregulated genes expression involved in T-cell activation, survival, cytokine signaling and T-cell toxicity (e.g., IL2RA, IL2RB, PRDM1, ICOS, CD40), and prevent tumor growth for 36 days^87,88^. Besides, immunomodulating and antigen-presenting function of engineered macrophages also help with T-cell activation in TME.

In another research, engineered microglia as the source of IL-15 was recruited to the tumor site. Researchers used rAAV2 (recombinant AAV serotype 2) carrying IL-15 to modify microglia. IL-15 (interleukin) promotes a pro-inflammatory phenotype of microglia and the cytotoxic activity of natural killer (NK) cells in TME, which also promote the production of IFN-γ, and counteracted tumor growth^89^. IL-12 has a similar therapeutic effect as an immunomodulator, but requires local administration to reduce systemic toxicity^90^. Expression of IL-12 by macrophages at subcutaneous tumor sites can improve the function of IFN cascade and activate T cells, slow tumor growth and prolong survival^91^. The therapeutic effect of immunomodulatory factors is confirmed. The use of macrophage carriers can improve the targeting and persistence of such therapies.

In the context of suicide gene/prodrug therapy, a novel non-viral gene vector technique based on light treatment was used to mediate the transfection of CD (cytosine deaminase) gene to macrophages in vitro. Transfected NR8383 cells could express CD with F98 glioma cells in the presence of 5-FC (5-fluorocytosine), a nontoxic precursor to 5-FU (5-fluorouracil). Because of the multi-drug resistance of NR8383 macrophages, transformed 5-FU is significantly more toxic to tumor cells than macrophages, allowing them to survive and consistently express CD^92,93^. This study shows high potential; however, further research is required to construct human-derived drug-resistant macrophage vectors for suicide gene therapy.

## Macrophages as therapeutic drug carrier

In spite of the emergence of new chemotherapy or immunotherapy agents, passive delivered free drugs show limited efficacy because of poor diffusion into brain tumor tissue^81,94^. The transport of free drug is affected by blood–brain barrier, uneven tumor vasculature and the pH of the tumor microenvironment^95^. Tumor-targeted cell-based delivery system exploit neural stem cells, mesenchymal stem cells, and monocyte/macrophages^96^, among which monocytes have the widest source, and this makes monocytes/macrophages an ideal vehicle for drug delivery^97^. In this way, the pro-tumor microenvironment was exploited as cellular “Trojan Horses” against malignances^94^.

To deliver chemotherapy drugs by macrophages, the most significant aspect is to avoid the toxicity of chemotherapy drugs to the carrier. Wang et al. built ND-PG-RGD-DOX (doxorubicin) with good aqueous solubility in physiological media, and it binds to the integrin receptor avβ3 that is overexpressed on the surface of multiple cells. Nano-DOX was sequestered in the lysosomal compartment which may mechanistically contribute to monocytes’ tolerance to the drug. Monocyte took up the Nano-DOX and maintained good viability for at least 48 h. Upon recruitment to the tumor microenvironment, monocytes are induced by GBM cells to differentiate and release more Nano-DOX than in peripheral blood. The drug delivery and tumor-killing efficacy of this method has been demonstrated in orthotopic GBM xenografts^95^. Notably, Monocytes release Nano-DOX in the periphery no slower than in the tumor, but the exocytosis of Nano-DOX from monocytes is calcium channel dependent. Perhaps the combination with calcium channel blockers (e.g., verapamil) may limit the non-specific release of the drug in the periphery^95^.

Liposomes are also employed to isolate the drug and reduce toxicity to carrier cells. Using dipalmitoyl phosphatidylserine (DPPS) as a “eat me” signal, paclitaxel (PTX)-loaded liposomes were phagocyted by BV2 microglia. Microglia then cross the blood–brain barrier and deliver the drug to tumor cells via extracellular vesicles and microtubules. Owing to its high targeting performance and natural accumulation in gliomas, this cell remedy requires far less dose of PTX, and has superior antitumor effect than sole PTX-liposome or PTX therapy^98^. In addition, the increase in CD86/CD206, TNF-*α*/IL-10, and CD8/FoxP3 ratios of TAM after administration also suggested that this regime could modulate the tumor microenvironment toward a pro-inflammatory phenotype.

Conventional chemotherapy drug TMZ also faces under-delivery for GBM therapy. Mia et al. have developed a noninvasive gut-to-brain oral drug delivery system dependent on macrophages. TMZ prodrug was encapsulated in nanoparticle (NP) with β-glucans using a GSH-responsive disulfide-containing linker and were phagocytosed in situ by resident macrophages in the intestinal tract, and then delivered to brain tumor site via the lymphatic and circulatory system. Bisulfide bonds within the prodrug NPs make sure that the drugs are only released in GSH (glutathione)-overexpressing tumor microenvironment^99^. The TMZ that can be delivered to the intracerebral tumor tissue using prodrug NPs is five times more than that using free TMZ with some present in the liver and minimal amounts in other major organs. This treatment improved survival and reduced weight loss in mice.

Some non-chemotherapy approaches require targeted delivery of therapeutic agents. Photothermal therapy (PTT) is to induce rapid heating in tumors via gold–silica nanoshells (AuNS) mediator, which are loaded into NR8383 macrophages for delivery to the tumor site and absorbs near-infrared light to produce a therapeutic effect^100,101^. Photodynamic therapy (PDT) has been implemented for GBM therapy. Its effect depends on photosensitizer (PS), light and oxygen in the irradiated tumor position. Conjugated polymer nanoparticles (CPNs), as a photosensitizer, were transported by macrophages infiltrated GBM in the U87 and GL261 bearing mice. CPNs were not found to affect monocyte viability, and the using of macrophage vehicle shows superior delivery efficacy to using sole CPNs^102^. However, this study did not provide in vivo antitumor experimental results.

## Chimeric antigen receptor-macrophage therapy

Although CAR T-cell therapy has demonstrated effectiveness and enhanced targeting for hematologic tumors, the recruitment of T cells to GBM tumor sites is limited by multiple mechanisms, including the blood–brain barrier, T-cell deletion^103^, and T-cell sequestration^104^. While several approaches have been tried to increase the infiltration of CAR-T cells in solid tumors, including GBM^105,106^, the suppressive tumor microenvironment could also render T-cell anergy^107^ and dysfunction^108^. As a result, creating CAR-T cells suitable for GBM treatment remains a challenge^109^. However, macrophages, as a crucial part of innate immune system, efficiently infiltrate into tumors, phagocyte and deplete abnormal cells, and ingest and present antigens to T cells^110^. These properties of monocytes/macrophages suggest expressing CAR on macrophages platform can enhance targeting and serves as a potential method of immunotherapy.

CAR-macrophage therapy is a promising area of research that has shown potential in treating various types of tumors. While very few studies have targeted GBM, one impressive method for extracranial tumors was developed by Klichinsky et al. Their CAR is based on a replication-incompetent chimeric adenoviral vector (Ad5f35), which persistently expressed CARs in macrophages and did not affect other functions. The receptor structure consists of a HER2 antigen-binding domain and an intracellular CD3ζ base domain which activate the phagocytosis of cells^111^. CAR-macrophages became pro-inflammatory (classically activated) phenotype after the stimulation of Ad5f35 vector, which could remodel suppressive TME. What’s more, as professional antigen-presenting cells, CAR-macrophages cross-present tumor antigens and activate T cells. Their CAR-macrophages extended the median survival time of SKOV3-burdened mice from 63 days to 88.5 days^112^ and CAR-M constructed using their strategy is in clinical trials (NCT04660929). CAR-macrophage therapy introduces a new means of exploiting adenovirus for tumor treatment and to some extent reshapes the immune microenvironment of tumors, suggesting that this therapy is of great significance.

Except for conventional CAR cell treatment that requires isolation, genetic modification and then reinfusion, there are new techniques to genetically engineering the intracavitary macrophages in situ to express CAR. Chen et al. constructed CD68 promoter-driven anti-CD133 CAR plasmids (pCARs) encoding the CD3ζ intracellular costimulatory domain, and used nanoporter (NP)–hydrogel superstructure for locoregional induction of CD133-specific CAR-MΦs in tumor resection cavity. Surrounding macrophages can be effectively transfected to express CARs, and then phagocytizes CD133 marked glioma stem cells and suppresses tumor growth and recurrence^113^. Locally engineered CAR-M cells exhibit a pro-inflammatory phenotype with only minor systemic side effects. This research provided us a new stand in CAR-macrophage therapy.

## Conclusion and future perspectives

TAMs play a crucial role in the tumor immune response due to their phenotypic diversity, which can result in either tumor-promoting or tumor-suppressing effects. However, the success of therapies targeting TAMs relies on our comprehensive understanding of their properties. A deeper understanding of TAMs' role in tumor development can facilitate the identification of more precise therapeutic targets and reduce tumor resistance to treatment. Conventional treatments and immunotherapy can be evaded by tumor cells through evolution^114^ and immune editing^115^. Studies have shown that TME components, particularly TAMs, co-evolve with tumors, making it challenging to target all macrophages crudely, leading to therapeutic resistance^116^. Potential strategy to overcome this challenge is to design more precise treatments based on the various TAM components' functions. In addition, combining TAM clearance with engineered macrophage introduction can utilize the competitive effect between cell populations to evade treatment resistance^112,117,118^. These strategies highlight the need to comprehend TAMs' mechanism of action and drugs.

During TAM polarization, macrophages undergo a complex process that involves metabolic changes and changes in the expression level of human leukocyte antigen (HLA) and CCL molecules. However, our understanding of this process is not yet complete. A study has identified the temporal changes of some M2-like polarization-associated molecules (such as MEK/ERK, peroxisome proliferator-activated receptor (PPARγ)) after treatment with IL-4^119^, and therapies have been designed to target these signaling pathways. Gradient changes in several immune molecules (e.g., chemokine and major histocompatibility antigen) during the development of GBM have also been identified^120^. As a result, a deeper understanding of macrophage-related temporal changes in both intrinsic and engineered macrophages will help us gain a more specific understand of the detailed mechanisms of TAM-related therapy and develop better treatment methods.

Genetically engineered macrophage-based platforms can reduce the impact of the unique GBM tumor microenvironment on exogenous gene vectors^121^. However, conventional methods for modifying immune cells, such as T cells and NK cells, are not effective for monocytes/macrophages and their progenitors. Some intrinsic mechanisms of macrophages, such as restriction factors^122^ and the lack of corresponding receptors on the surface^123^, limit the function of commonly used viral vectors. Elaborate adenovirus may provide us with a way to stably express the desired gene in macrophages. Modified adenovirus recognizes a wider range of cellular markers than the commonly used coxsackie and adenovirus receptors^124,125^. For example, the Ad5/F35 chimeric virus has been used in preclinical and clinical studies for viral therapy of hematologic diseases as well as CAR-macrophage therapy because it recognizes the CD46 marker on macrophages and can effectively transduce them^126^. In addition to modified adenoviruses, modified lentiviral vectors which resist to certain restriction factors are also capable of expressing exogenous genes in monocytes and macrophages^127^. As for in vivo macrophage modifying, some AAV vector for gene therapy (such as AAV9) are able to cross the blood–brain barrier and can be used in combination with exosomes to enhance infection of TAM^84^. Several nano- or physical methods for transfection of macrophages using non-viral vectors are also under investigation^93,113^, although the persistence of these vectors still needs to be improved. To increase the density of expression vectors in the tumor microenvironment, we can also use oncolytic viruses that can replicate specifically in the tumor^128^. It is interesting to note that the vector used to modify macrophages can also affect their phenotype. Adenovirus and certain non-viral vectors can cause a shift in macrophages towards a pro-inflammatory phenotype^112,113^, while lentiviruses may not alter macrophage phenotype or lead to a pro-tumor phenotype^91,129^. The mechanism underlying these effects requires further investigation. Ultimately, the choice of vector depends on factors such as safety, efficiency, impact on macrophages, and potential toxicity of the gene being expressed.

The appropriate cell sources are crucial for macrophage therapy. However, monocytes, the primary source of macrophages, are scarce in peripheral blood, which makes it challenging to harvest enough macrophages for therapy. In such cases, induced pluripotent stem cells (iPSCs) can be used to produce macrophages with therapeutic effects, such as CAR-macrophages^129,130^. Moreover, self-renewing hematopoietic stem/progenitor cell (HSPC) are also potential sources of macrophages. Under certain conditions, such as bone marrow transplantation with CSF-1R blockade treatment, circulation-derived myeloid cells (CDMC) can replace microglia in brain tissue and potentially serve as a source of macrophages for therapy^117^. However, further validation of this approach is needed under tumor conditions. Although processing the human hematopoietic system is complex, based on the tumor tropism of macrophages, it is hypothesized that delivering HSC-derived macrophages to tumor tissue may not be as demanding as delivery to brain tissue^83^.

In terms of CAR therapy, several targets have been explored for GBM therapy, including interleukin-13 receptor alpha 2 (IL13Rα2), EGFRvIII, HER2, CD70, B7-H3, and others^86,131–133^, although many of them do not produce decisive outcomes in CAR-T therapy due to immunosuppressive TME, antigen drift or downregulation and heterogeneity of solid tumors. Various approaches, such as the introduction of immunosuppressants, chemokines, and increased types of CARs or CAR-T cells, have been tried to overcome these challenges^105,133,134^. CAR-macrophage therapy shows promise in overcoming several challenges that have hindered the application of CAR-T cells in GBM. Unlike CAR-T cells, CAR-macrophages have superior tumor infiltration capabilities and work by not only directly killing tumor cells but also stimulating the immune system, remodeling the TME, and presenting antigens^113,134^. As a result, CAR-macrophages may be less affected by tumor heterogeneity and downregulated CAR targets, which can hinder the effectiveness of CAR-T therapy^135^. However, more research is needed to fully understand the mechanisms underlying CAR-macrophage therapy. In addition, compared to CAR-T therapy, CAR-macrophage therapy has shown relatively low systemic toxicity in preclinical studies. It is hypothesized that macrophages downregulate migration-associated receptors (CCL2, CCL5) in the hypoxic TME, which may trap recruited CAR-macrophages in the tumor site and reduce systemic toxicity^136^. Moreover, local treatment strategies following routine surgeries for GBM may also help to reduce systemic toxicity^113^.

Despite the potential benefits of CAR-macrophage therapy, there are unique challenges that need to be addressed. One major challenge is maintaining the pro-inflammatory phenotype of macrophages while avoiding their pro-tumor functions. In addition, many of the current CAR-M treatments utilize the same CAR structure as in T cells, which may not be optimal for achieving both tumor cell killing and TME regulation. To overcome these challenges, it may be necessary to design CARs that can more effectively activate multiple functions of macrophages^137^. In conclusion, further research is needed to explore the specific mechanism of CAR-M therapy, investigate the temporal changes in the immune microenvironment after administration, and develop the optimal design of CARs suitable for macrophages.
